# How Authoritative Media and Personal Social Media Influence Policy Compliance Through Trust in Government and Risk Perception: Quantitative Cross-Sectional Survey Study

**DOI:** 10.2196/64940

**Published:** 2025-01-20

**Authors:** Hua Zhang, Cheng Yang, Xiuxian Deng, Chunyan Luo

**Affiliations:** 1 Guangxi Minzu University Nanning China

**Keywords:** paradox of trust, risk perception, trust in government, policy compliance, pandemic, authoritative media, social media, China

## Abstract

**Background:**

Previous studies on public compliance with policies during pandemics have primarily explained it from the perspectives of motivation theory, focusing on normative motivation (trust in policy-making institutions) and calculative motivation (fear of contracting the disease). However, the social amplification of a risk framework highlights that the media plays a key role in this process.

**Objective:**

This study aims to integrate the motivation theory of compliance behavior and the social amplification of risk framework to uncover the “black boxes” of the mechanisms by which normative motivation and calculative motivation influence public policy compliance behavior through the use of media.

**Methods:**

During the COVID-19 pandemic, we conducted a web-based survey of 2309 Chinese citizens from December 4, 2022, to March 21, 2023. We treated the public’s policy compliance behavior during the pandemic as the dependent variable. Media use, specifically the use of authoritative media and “we-media,” that is, personal social media accounts and media platforms operated by individuals, was set as the independent variable. Trust in government, representing normative motivation, and risk perception, representing calculative motivation, were included as mediating variables. A structural equation model was constructed and analyzed using Stata.

**Results:**

First, the mediation effect of trust in government indicates that the use of authoritative media can enhance trust in government, which significantly increases individuals’ policy compliance behavior (β coefficient=0.108, 95% CI 0.080-0.135; *P*<.001). Second, the mediation effect of risk perception shows that the use of we-media heightened individual risk perception, thereby significantly enhancing policy compliance behavior (β coefficient=0.012, 95% CI 0.002-0.021; *P*=.02). Third, the study revealed the “paradox of trust”: the chain mediation effect in which authoritative media increased trust in government and reduced risk perception, ultimately decreasing policy compliance behavior (β coefficient=–0.005, 95% CI –0.009 to –0.001; *P*=.008).

**Conclusions:**

By combining the motivation theory of compliance behavior with the social amplification of risk framework in risk communication, we found that trust in government, as a normative motivation, operates through authoritative media, while risk perception, as a calculative motivation, promotes compliance behavior through we-media. In addition, in major crises, the public’s use of authoritative media can lead to the paradox of trust: on the one hand, trust in the government increases policy compliance; on the other hand, this trust reduces risk perception, thereby decreasing compliance behavior. Authoritative institutions need to balance providing authoritative information with maintaining the public’s risk perception.

## Introduction

### Background

During the early stages of the COVID-19 pandemic, the Chinese government implemented strict control measures, such as mask-wearing mandates, compulsory nucleic acid testing, and vaccination programs, which effectively curbed the spread of the virus. However, in late December 2022, the government relaxed these stringent policies. Established experience has highlighted the importance of public compliance with health policies in containing the virus [1,2]. It is perhaps more crucial to increase citizens’ willingness to comply with proactive prevention policies than to formulate these policies [3]. If citizens do not adequately follow epidemic control measures, governmental efforts may be wasted [4]. Current research on compliance with public health policies mainly adopts 2 perspectives from motivation theory. The normative motivation perspective emphasizes that trust in policy-making institutions helps increase citizens’ willingness to adhere to public health interventions [5], whereas the calculative motivation perspective argues that compliance behavior stems from the fear of contracting the disease [1,6].

During a pandemic, the public is in a state of “information hunger” and often actively seeks various sources of information to fill their “information vacuum” [7]. The role of information disseminated through different media in shaping psychological processes and individual behaviors has been widely studied across various fields [8-11]. The social amplification of risk framework (SARF) emphasizes that social media, as well as traditional print and broadcast news media, can serve as “social amplification stations,” shaping the public’s risk perception [12]. Within this framework, countries have used diverse media to promote the importance and urgency of COVID-19 prevention measures, such as vaccine administration, mask-wearing mandates, and home isolation [13,14]. Therefore, understanding the crucial role of different media in promoting policy compliance should be a key focus for future research [15].

The significance of this study lies in the following. This study was conducted during the COVID-19 pandemic, a unique context that holds substantial research value. Media usage habits and their behavioral outcomes often shift due to the specificity of the situation, making conclusions drawn from general social contexts not necessarily applicable to pandemic scenarios [16]. The COVID-19 pandemic provides a distinctive natural experimental condition for investigating the role of media in public health emergencies, offering deeper insights into the dynamic mechanisms of media in risk communication and behavioral influence compared to studies conducted in general social contexts.

The innovation of this paper lies in several key aspects. In the past, authoritative media often played the primary role of information disseminators and communicators. However, with the rise of the internet and “we-media,” that is, personal social media accounts and media platforms operated by individuals, this traditional model is gradually being disrupted. Unlike previous studies on risk communication effects that generally lacked attention to media diversity [6] or those that separately explored the effects of risk perception and trust in government on policy compliance behavior [17,18], we conducted a more in-depth and comprehensive exploration. Specifically, we categorized media into authoritative media and we-media and incorporated them, along with trust in government (representing normative motivation) and risk perception (representing calculative motivation), into a structural equation model (SEM) for analysis. This approach allows us to unveil the “black boxes” of how trust in government and risk perception operate between the use of different media and public policy compliance behavior, thereby providing a more comprehensive investigation of how various media influence public compliance with health policies. This multidimensional analysis not only enriches existing research perspectives but also offers more precise and effective guidance for potential public health crises in the future.

### Literature Review

#### Motivational Perspectives on Compliance Behavior

##### Overview

Previous research on compliance with public health policies during pandemics primarily explains behavior from the perspectives of normative motivation and calculative motivation within motivation theory [1,3]. Normative motivation focuses on individuals’ intrinsic adherence to social responsibility and moral norms [19], while calculative motivation emphasizes individuals’ cost-benefit analysis during decision-making [20]. Although these 2 motivations largely explain public behavior, in practice, they often intertwine and collectively influence individuals’ compliance decisions.

##### Normative Motivation: The Role of Trust in Government

Normative motivation involves the internal moral beliefs, values, and social norms that guide individual behavior, primarily stemming from trust in the government [17,21], a sense of social responsibility [1], social interaction [20], and social and peer pressure [19].

The characteristics of trust in government, such as policy legitimacy and institutional trust, are key sources of individuals’ normative motivations [22]. China effectively curtailed the pandemic through “rapid and decisive” prevention measures, which enhanced public trust in the government [23]. Strong trust in government facilitates public compliance with government policies and the implementation of these policies [5]. The positive effect of trust in government on policy compliance has been demonstrated not only in China but also in Italy, where research indicates that during the COVID-19 pandemic, regions with high trust in the government saw the public actively responding to government calls and reducing their outdoor activities [24]. In addition, a study in Sweden found that higher public trust in the government correlated with greater self-efficacy, leading individuals to be more inclined to follow government strategies for managing public health risks, thereby also helping to mitigate the pandemic [21]. Increased public trust in government can substantially enhance the effectiveness of governmental management during a pandemic, ensuring smooth policy implementation [25,26]. Some studies have found a negative correlation between government trust and compliance behavior, a phenomenon referred to as the “paradox of trust.” Three theoretical frameworks can be identified within this discourse: the “chain reaction of confidence,” the “paradox of support,” and the “paradox of confidence” [27]. Furthermore, a “trust, confidence, and cooperation” model has been proposed in the context of risk management [28].

With regard to the COVID-19 pandemic, the basic premise of the trust paradox is that the pandemic is viewed as a source of psychological stress, with trust serving as a psychological defense mechanism that can mitigate the complexities and uncertainties often present during a crisis [27,29,30]. Consequently, public trust in the government’s ability to manage the pandemic may lead to a reduction in individual protective behaviors because individuals perceive the government’s measures to be sufficient, thereby decreasing their motivation to take additional protective actions [31]. Research has also indicated that in environments where public trust in government is low, individuals may feel a stronger need to engage in personal protective behaviors to alleviate their fear, which can, in turn, enhance support for pandemic containment efforts [32]. These varying manifestations of the paradox of trust profoundly reveal the multidimensional complexity of the relationship between trust and compliance behavior. This finding underscores the necessity for more in-depth exploration in this field. Therefore, this study further analyzes the intrinsic mechanisms underlying the complex relationship between trust and compliance behavior, aiming to provide richer theoretical support and empirical evidence for research in this area.

##### Calculative Motivation: The Impact of Risk Perception

Unlike normative motivation, which focuses on individuals’ intrinsic moral beliefs, calculative motivation emphasizes individuals’ weighing and evaluation of the consequences of their actions. Within this theoretical framework, prospect theory, deterrence theory, and protection motivation theory are particularly emphasized. Prospect theory reveals that people are influenced by psychological biases, making them likely to take decisions based on the similarity of events to known samples and the availability of relevant information in memory [33]. Deterrence theory posits that strict policies and enforcement will enhance the public’s motivation to comply with policies [34]. Protection motivation theory suggests that high levels of risk perception can influence the public’s intention to take protective measures [35]. This study mainly uses risk perception as an entry point to investigate public compliance with policies.

Protective motivation theory offers a valuable framework for understanding public policy compliance behavior during pandemics. Within this theory, risk perception refers to people’s subjective judgment of the characteristics and severity of a specific risk, which is the initial reaction individuals have when a crisis occurs [36]. The consequences of risks can directly impact the morbidity and mortality rates of populations considered vulnerable. Although risk perception can have negative effects, such as causing social panic and increasing the costs of government-public cooperation [37], many studies have pointed out that risk perception can also produce positive effects. Risk perception plays a critical role in crisis disasters and in encouraging citizens to cooperate with government preventive and mitigation measures [38]. The positive relationship between government intervention and disaster prevention and control is built on the public’s active cooperation with government interventions [37,39]. Specifically, in the context of epidemic spread, studies have found that lower COVID-19 risk perception is associated with lower compliance with social distancing measures [18]. Therefore, public awareness of risk is a key factor in promoting compliance with protective behaviors.

#### Differences in Media Use During Pandemics

##### Overview

Traditional communication theories assert that risks and risk events primarily flow through 2 main communication networks: the news media and interpersonal communication networks [12]. During a pandemic, the public relies on various media to stay updated on the latest developments and necessary self-protection measures [40-42]. Among these media, television news, newspapers, and official news websites became the primary sources for the public to obtain information related to the COVID-19 pandemic. Simultaneously, social media platforms such as Facebook and Twitter (subsequently rebranded as X) were widely used by the public for information due to their convenience and interactivity [16,31,43,44]. Research shows that traditional (authoritative) media tend to support government and official viewpoints, paying little attention to the dissemination of differing opinions and often providing a less polarized view of events. By contrast, emerging we-media platforms, characterized by multiple topics and platforms, diverse themes, and varying perspectives, facilitate the easy spread of extremist viewpoints [45]. This study, combining its unique characteristics with existing literature [45-47], categorizes the former as authoritative media (also known as mass media or traditional media) and the latter as we-media (also known as new media or online media). This classification can help us better understand the characteristics and patterns of information dissemination and formulate more precise and effective information communication strategies.

##### Authoritative Media

The official websites of governments and authoritative institutions are considered the primary sources of authoritative information for the public, acting as the main media that directly affect the public’s risk perception [48,49]. Official media often use expert opinions to enhance their authority and persuade the public, using methods such as framing effects or agenda setting. These media primarily operate on a “we-publish-you-read” model [50]. Authoritative media typically provide the latest policy updates, professional guidance, and important announcements, making them crucial channels for the public to obtain reliable information [51]. With their long-established “opinion guidance” capabilities, they gain public recognition and trust [52]. These authoritative media are responsible for providing accurate, unbiased, and reliable information and preventing the spread of misinformation [46,53].

##### We-Media

The rapid development and widespread adoption of the internet have ushered users into a brand new era of we-media. In this we-media age, all users have a microphone and function as both creators and disseminators of public opinion [54,55]. On the one hand, the we-media formed by family and friends provide references for verifying viewpoints. In a broad sense, this involves sharing passed-on perspectives, which can either amplify or diminish the information [12]. On the other hand, we-media represented by social media platforms such as TikTok offer more personalized content. These platforms use algorithms to recommend relevant content to users and have more interaction-enabled features, allowing users to participate in content creation and discussion. Research has shown that social media played a substantial role in spreading misinformation about COVID-19 [56,57]. Misinformation can lead the public to doubt the severity of a pandemic and the effectiveness of prevention measures, thereby reducing the likelihood of following these measures [18,46].

### Theoretical Model and Hypotheses

On the basis of the reviewed literature, we constructed a theoretical model that includes the use of media, normative motivation, and calculative motivation related to compliance with public health policies. Specifically, we propose 3 hypotheses and corresponding supplementary hypotheses to examine the process by which the public’s use of authoritative media and we-media influences individual policy compliance behavior through trust in government and risk perception.

Research on COVID-19 has found that authoritative media serve as an important channel for the public to obtain reliable information [51], while we-media are characterized by greater diversification and personalization [16,45]. Moreover, the public’s trust in government can have varying effects on policy compliance behavior [17,31]. On the basis of the characteristics of authoritative media and we-media, we propose the following hypotheses:

- H1a: The use of authoritative media enhances individual policy compliance behavior by increasing trust in government.
- H1b: The use of we-media decreases individual policy compliance behavior by lowering trust in government.

Kasperson et al [12] proposed the SARF, which suggests that the media can act as “social amplification stations,” amplifying or attenuating the public’s risk perception. Protective awareness, stakeholder engagement, and risk awareness can positively influence individuals to take protective measures, with risk perception playing a partial mediating role [58]. Therefore, we propose the following hypotheses:

- H2a: The use of authoritative media decreases individual policy compliance behavior by lowering risk perception.
- H2b: The use of we-media increases individual policy compliance behavior by raising risk perception.

Finally, we believe that there is a chain mediation effect of trust in government and risk perception between media use and policy compliance behavior. Members of the public exposed to authoritative media are more likely to trust the government [59]. While some studies found that the use of we-media affects individuals’ trust in government [59,60], others found no effect [61]. Moreover, previous studies have found that public trust in government can lower risk perception, leading to reduced compliance with policies [31]. In addition, in environments with low government trust, the public may show greater support for pandemic containment measures [32]. These varying phenomena highlight the “paradox of trust,” suggesting the need for further exploration of the complex relationship between government trust and policy compliance.

Consequently, this study constructs a dual mediation model aimed at revealing how different media influence risk perception through trust in government, ultimately shaping attitudes toward policy compliance. Building on prior research, we propose the following hypothesis:

- H3a: The use of authoritative media positively influences trust in government, prompting risk perception to negatively affect policy compliance behavior.

Furthermore, we posit that due to the diversity and personalized content of we-media, the use of we-media may lower public trust in the government, thereby affecting risk perception from a dual perspective, subtly influencing individual policy compliance behavior. Thus, we propose another innovative hypothesis:

- H3b: The use of we-media negatively impacts trust in government, resulting in a positive effect of risk perception on policy compliance behavior.

Interestingly, the “paradox of trust” previously revealed in studies could be triggered if either hypothesis H3a is validated, indicating that the use of authoritative media indeed enhances government trust and leads to a negative influence of risk perception on policy compliance behavior, or hypothesis H3b holds true, suggesting that the use of we-media weakens government trust as anticipated, resulting in a positive influence of risk perception on policy compliance behavior. This phenomenon is not only thought provoking but also ignites our enthusiasm and expectation for exploration and validation.

**Figure 1:**
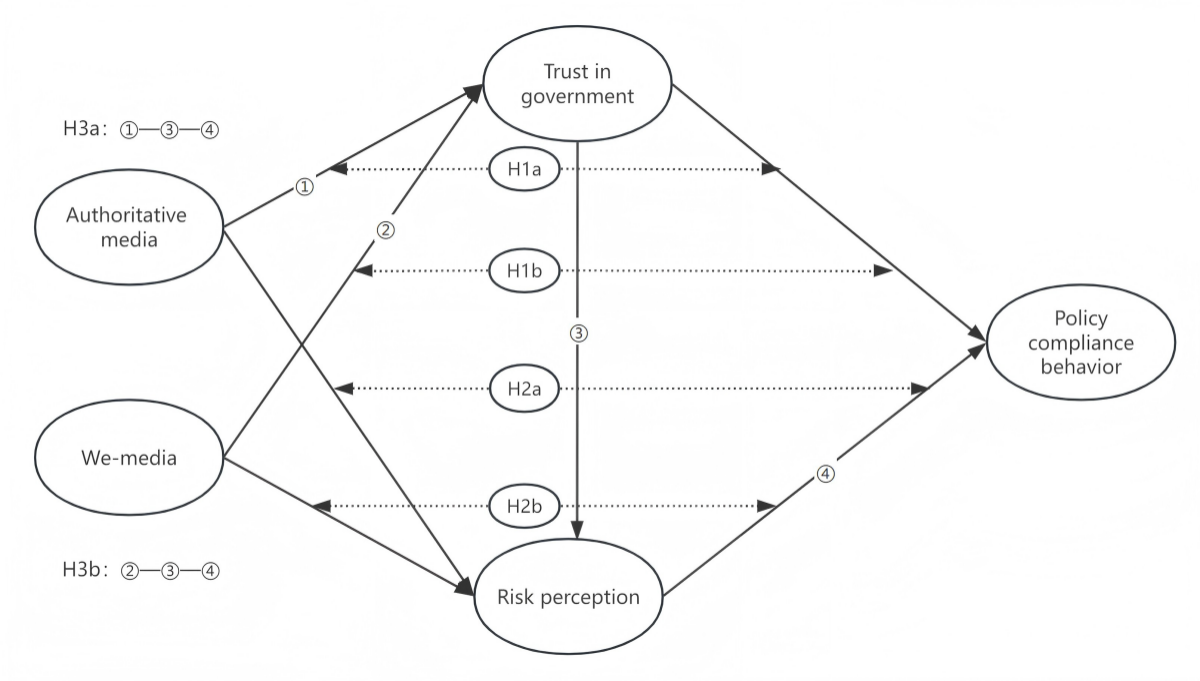
Hypothetical model of the theory.

On the basis of the hypotheses proposed based on the aforementioned theoretical framework, we constructed the theoretical model diagram shown in Figure 1.

## Methods

### Data Collection

This study used purposive sampling and snowball sampling methods to collect samples through web-based surveys, with the sample collection period spanning from December 4, 2022, to March 21, 2023. During the questionnaire design phase, we used the professional platform Wenjuanwang to construct the questionnaire framework. To ensure the accuracy and clarity of the questionnaire items, we conducted an extensive literature review and consulted experts from relevant fields. Building on this foundation, we drafted the initial content of the questionnaire.

Subsequently, to further optimize the questionnaire, we conducted a small-scale pilot survey to evaluate the feasibility of the questionnaire items and assess respondents’ understanding. On the basis of the pilot survey results, we made necessary adjustments and finalized the questionnaire version. We then promoted the survey through advertisements on social media platforms, specifically WeChat, and recruited participants via web-based snowball sampling. Although the response rate for web-based sample surveys can be low, web-based surveys proved to be a feasible method for conducting research during the pandemic when social distancing and epidemic prevention measures were in place because they are accessible via smart devices and do not have location constraints.

In total, we collected 3690 completed questionnaires. After completing the data cleaning process, we conducted a common method bias (CMB) test to ensure the validity of the data collection. The detection of CMB typically involves exploratory factor analysis (EFA) and the Harman single-factor test. If the first unrotated common factor accounts for >50% of the variance, it may indicate the presence of CMB. The results of our EFA revealed that the variance explained by the first common factor was 23.9%, thereby rejecting the null hypothesis of the Harman single-factor test and indicating that there was no CMB.

### Statistical Methods

After completing the data collection, we used Stata (version 16; StataCorp LLC) to perform statistical analysis. First, the basic characteristics of the data were described, followed by the construction of an SEM. The model aimed to explore the relationship between public media use and adherence to public health policies in depth.

### Variable Measurement

The questionnaire included items on policy compliance behavior, media use, trust in government, and risk perception, using a 5-point Likert scale for the responses. It also included demographic questions about the respondents’ gender, education level, residence type, and annual family income (refer to the Results section for details).

### Policy Compliance Behavior

The study used policy compliance behavior as the dependent variable. On the basis of the protection guidelines issued by the National Health Commission of China in August 2021 and existing research [6,62], the questionnaire asked respondents, “How frequently do you follow the following personal protective measures in your daily life?” This question evaluated 4 measures: wearing a mask, washing hands, maintaining a 1-meter distance in public places, and getting vaccinated. Responses ranged from 1=*never* to 5=*always*.

### Media Use

The study used “media use” as the independent variable. Information from authoritative media typically has official and authoritative endorsement from the government, whereas we-media contain more diverse and liberal content. Referring to existing literature [10,46], the questionnaire asked respondents, “How frequently do you use the following media to obtain information in your daily life?” This question assessed the frequency of using various media, including government television news, business websites and news apps (eg, Sina Weibo), health agency websites (eg, expert sources), family and friends, short video apps (eg, TikTok), and social media (eg, QQ and WeChat). Responses ranged from 1=*never* to 5=*always*.

We conducted an EFA on media use (refer to the Results section for details).

### Trust in Government

The study used trust in government and risk perception as mediating variables. Trust in government refers to government credibility [63]. When government credibility is high and recognized by the public, the trust in the government or system increases [64]. Therefore, the study referred to the measurement methods of government credibility by Grimmelikhuijsen and Meijer [65] and Ma and Sun [66], combined with the characteristics of Chinese public trust in the government, to measure trust in government by evaluating public satisfaction with specific government actions. The measures included “Your satisfaction with the transparency of government information” and “Your satisfaction with the timeliness of government information disclosure.” These indicators capture the public’s direct perceptions of the government’s ability to disclose information, thereby indirectly reflecting their overall evaluation of government credibility. Scores ranged from 1=*very dissatisfied* to 5=*very satisfied*.

### Risk Perception

The measurement indicators for risk perception typically include the severity of the event and its impact scope, with items such as “this event is very severe” and “the impact of this event is very broad” [67,68]. However, during a large-scale crisis, the impact on socioeconomic and personal daily life is especially substantial, making it difficult to accurately measure the heterogeneity of individual risk perception based solely on event severity and impact scope. Therefore, we drew on the research by Zhu et al [69], which uses the direct consequence of risk perception—a sense of security—to measure the cognitive level of risk. The questionnaire asked, “What is the source of your insecurity?” The options included fear of self or family members becoming infected, concern about the lack of material security, worry about employment issues during the public health crisis, and fear of being locked down or quarantined. Respondents rated their concerns from 1=*not worried at all* to 5=*very worried*.

### EFA and Reliability and Validity Testing

We conducted EFA using Stata. As policy compliance behavior, risk perception, and trust in government were measured using well-established scales from previous research, only media use underwent EFA. The initial factors were rotated using the Kaiser normalized maximum variance method. On the basis of the EFA results, we labeled factor 1 as “authoritative media use” and factor 2 as “we-media use.”

Further confirmatory factor analysis was conducted on each dimension of the scale. The fit indices for the measurement model were as follows: *χ*^2^_94_=674.1; *P*<.001; root mean square error of approximation=0.052; comparative fit index=0.961; Tucker-Lewis index=0.950; standardized root mean square residual=0.033; this indicated good overall model fit, supporting the validity and reliability of the measurement results. The Cronbach α values for each dimension of the scale ranged from 0.70 to 0.93, all exceeding the 0.55 threshold commonly used in social science research, and the overall Kaiser-Meyer-Olkin measure of sampling adequacy was 0.77. Factor loadings for each item ranged from 0.48 to 0.95, suggesting moderate to strong associations between the measured items and their respective latent factors. According to Stevens [70], a factor loading of 0.40 is sufficient for explanatory purposes, even with varying sample sizes. Furthermore, Steenkamp and Maydeu-Olivares [71] found that lower factor loadings can still be explanatory, particularly in cases of smaller sample sizes or more complex object of study.

### Ethical Considerations

All participants in this study provided informed consent. The study was approved by the Science Ethics and Safety Committee of Guangxi University for Nationalities (Protocol Number: 202410608021). All procedures conducted during the study strictly adhered to the ethical principles outlined in the Declaration of Helsinki (2013).

## Results

### Data Cleaning Process

**Figure 2:**
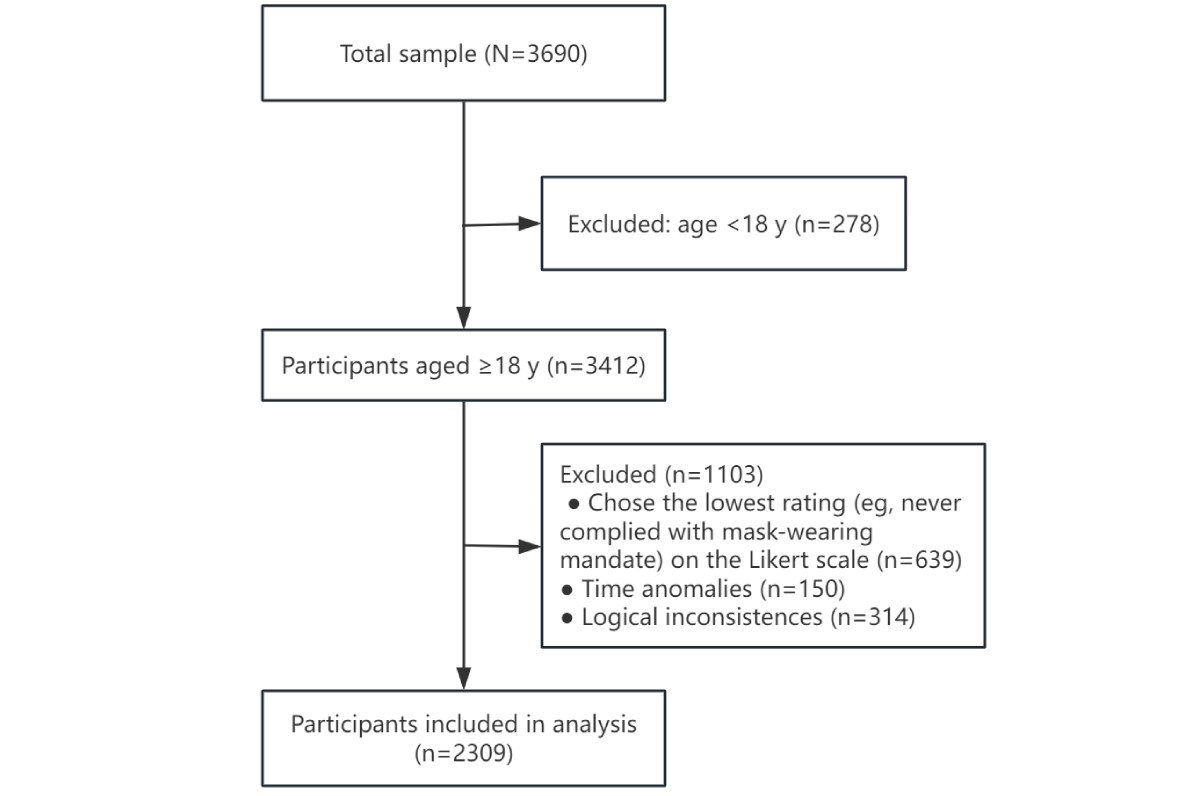
Flowchart of the data cleaning process.

We collected 3690 completed questionnaires in total. We used a dual review process to check data quality, focusing on outliers and logical inconsistencies. After cleaning the data, from the 3690 completed questionnaires, we ultimately obtained 2309 (62.57%) valid questionnaires. The flowchart of the data cleaning process is shown in Figure 2.

### EFA Results

The EFA results showed that the 6 items of media use were extracted into 2 factors. The items “government television news,” “business websites and news apps,” and “health agency websites” had higher loadings on factor 1, while “social media,” “short video apps,” and “family and friends” had higher loadings on factor 2 (Table 1). On the basis of existing literature [45-47] and the EFA results, we labeled factor 1 as “authoritative media use” and factor 2 as “we-media use.”

**Table 1:** Results of exploratory factor analysis.

Variable items	Factor 1	Factor 2
Government television news	*0.6396* ^a^	0.2452
Business websites and news apps (eg, Sina Weibo)	*0.6725*	0.2942
Health agency websites (eg, expert sources)	*0.4419*	0.2274
Short video apps (eg, TikTok)	0.2817	*0.5995*
Social media (eg, QQ and WeChat)	0.2828	*0.6426*
Family and friends	0.2732	*0.4293*

^a^Higher loadings are indicated in italics.

### Descriptive Statistics

The survey questionnaire included items on policy compliance behavior, media use, trust in government, and risk perception. Participants rated their responses on a Likert scale ranging from 1 to 5 (Table 2). The questionnaire also included demographic questions (Table 3).

**Table 2:** Descriptive statistics of variables: survey questionnaire responses rated on a 5-point Likert scale (n=2309).

Variables and categories			Scores, mean (SD; SE)
**Policy compliance behavior**			
	Wear a mask	4.38 (0.77; 0.01)	
	Wash hands	4.41 (0.72; 0.01)	
	Keep distance	3.98 (0.96; 0.01)	
	Vaccinate	4.65 (0.67; 0.02)	
**Authoritative media use^b^**			
	Government television news	3.90 (0.94; 0.01)	
	Business websites and news apps (eg, Sina Weibo)	3.76 (1.02; 0.01)	
	Health agency websites (eg, expert sources)	3.12 (1.11; 0.02)	
**We-media use^c^**			
	Short video apps (eg, TikTok)	3.99 (1.00; 0.02)	
	Social media (eg, QQ and WeChat)	4.09 (0.89; 0.02)	
	Family and friends	3.83 (0.94; 0.02)	
**Trust in government**			
	Timeliness of information	3.75 (0.89; 0.01)	
	Information transparency	3.69 (0.94; 0.01)	
**Risk perception^e^**			
	Fear of disease	3.24 (1.17; 0.01)	
	Fear of lack of security	3.02 (1.18; 0.01)	
	Worry about jobs	3.40 (1.21; 0.01)	
	Fear of isolation	3.26 (1.24; 0.01)	

^a^Likert scale ratings: 1=*never comply*, 2=*occasionally comply*, 3=*sometimes comply*, 4=*often comply*, and 5=*always comply*.

^b^Likert scale ratings: 1=*never use*, 2=*rarely use*, 3=*sometimes use*, 4=*often use*, and 5=*always use*.

^c^Likert scale ratings: 1=*never use*, 2=*rarely use*, 3=*sometimes use*, 4=*often use*, and 5=*always use*.

^d^Likert scale ratings: 1=*very dissatisfied*, 2=*somewhat dissatisfied*, 3=*neutral*, 4=*somewhat satisfied*, and 5=*very satisfied*.

^e^Likert scale ratings: 1=*not worried at all*, 2=*not very worried*, 3=*somewhat worried*, 4=*quite worried*, and 5=*very worried*.

**Table 3:** Descriptive statistics of variables: demographics (n=2309).

Variables and categories			Participants, n (%)
**Gender**			
	Women	1657 (71.76)	
	Men	652 (28.24)	
**Education level**			
	Below bachelor’s degree	950 (41.14)	
	Bachelor’s degree or higher	1359 (58.86)	
**Residence type**			
	City	774 (33.52)	
	Rural	1535 (66.48)	
**Annual income,** **CN ¥ (US $1=CN ¥** **6.7976** **)**			
	<20,000	779 (33.74)	
	20,001-100,000	830 (35.95)	
	100,001-200,000	506 (21.91)	
	>200,000	194 (8.4)	

### Estimation of Fit Indices

**Figure 3:**
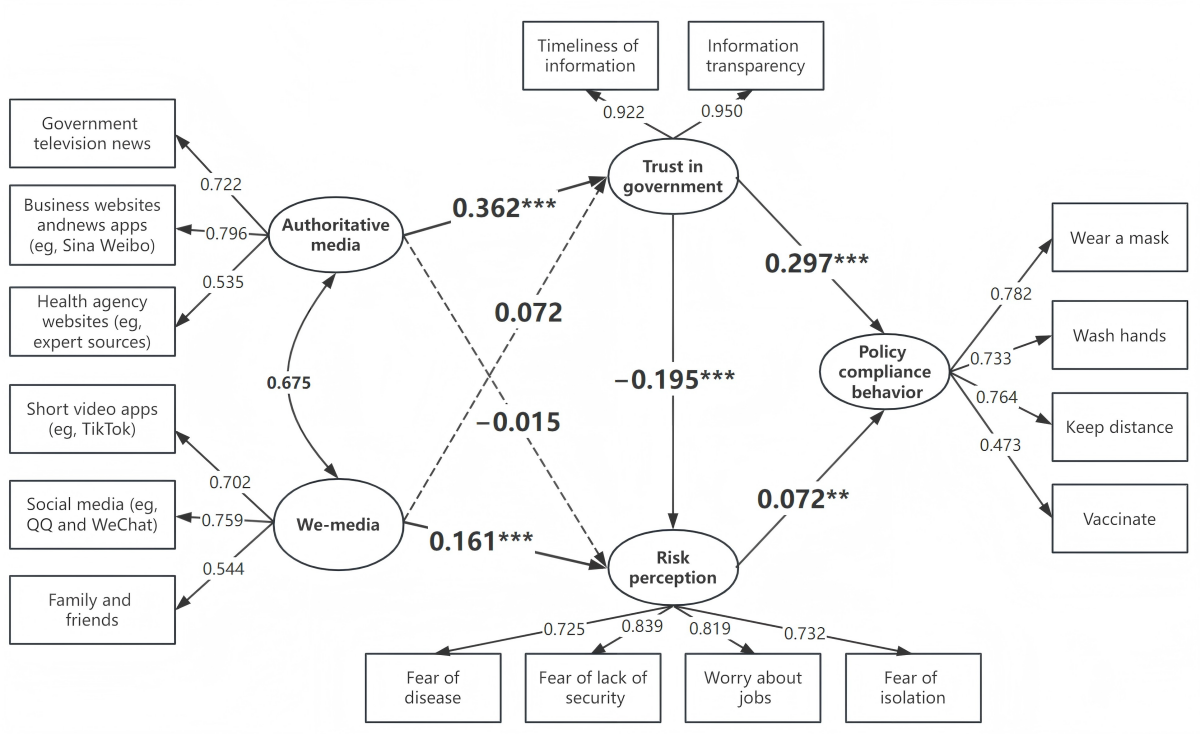
Structural equation model and path coefficient diagram. The solid lines indicate significant paths, while dashed lines indicate nonsignificant paths. **P<.01, ***P<.001.

Before conducting hypothesis testing, we used Stata to estimate the fit indices for the SEM. The results demonstrated satisfactory model fit (*χ*^2^_96_=722.3; *P*<.001; root mean square error of approximation=0.053; comparative fit index=0.958; Tucker-Lewis index=0.947; standardized root mean square residual=0.045). These findings indicate that the model meets acceptable standards and effectively fits the sample data without necessitating adjustments or respecification of the original model. Figure 3 presents the SEM along with its associated path coefficients (error terms for all endogenous variables are assumed but not depicted in Figure 3).

### Intermediate Path Test Results

Trust in government plays a significant positive mediating role in the path “authoritative media use→trust in government→policy compliance behavior” (β coefficient=0.108, 95% CI 0.080-0.135; *P*<.001; Table 4). The use of authoritative media effectively enhances public trust in the government, leading to increased compliance with policies. Therefore, H1a is supported. In the path “we-media use→trust in government→policy compliance behavior,” although the path coefficient is positive, the mediating effect does not reach statistical significance (β coefficient=0.021, 95% CI –0.001 to 0.044; *P*=.07). We-media use does not substantially affect public trust in the government, thus having no substantial impact on policy compliance behavior. Therefore, H1b is not supported.

**Table 4:** Intermediate path test results.

Paths	β coefficient (SE; 95% CI)	Critical ratio	*P* value
Authoritative media use→trust in government→policy compliance behavior	0.108 (0.014; 0.080 to 0.135)	7.70	<.001
We-media use→trust in government→policy compliance behavior	0.021 (0.012; –0.001 to 0.044)	1.84	.07
Authoritative media use→risk perception—policy compliance behavior	–0.001 (0.003; –0.007 to 0.005)	–0.36	.72
We-media use→risk perception—policy compliance behavior	0.012 (0.005; 0.002 to 0.021)	2.35	.02
Authoritative media use→trust in government→risk perception→policy compliance behavior	–0.005 (0.002; –0.009 to –0.001)	–2.64	.008
We-media use→trust in government→risk perception→policy compliance behavior	–0.001 (0.001; –0.002 to 0.000)	–1.52	.13
Authoritative media use→trust in governments→risk perception	–0.071 (0.012; –0.093 to –0.048)	–6.09	<.001
We-media use→trust in governments→risk perception	–0.014 (0.008; –0.030 to 0.001)	–1.78	.08

The path coefficient of risk perception between authoritative media use and policy compliance behavior is negative, but the mediating effect does not reach statistical significance (β coefficient=–0.001, 95% CI –0.007 to 0.005; *P*=.72). Authoritative media use does not significantly affect public trust in the government, resulting in no significant impact on policy compliance behavior. Thus, H2a is not supported. By contrast, the path “we-media use→risk perception→policy compliance behavior” is statistically significant and positive (β coefficient=0.012, 95% CI 0.002-0.021; *P*=.02). It shows that the public’s use of we-media enhances risk perception, promoting compliance with policies. Therefore, H2b is supported.

The chain mediation path “authoritative media use→trust in government→risk perception→policy compliance behavior” shows a significant chain mediation effect (β coefficient=–0.005, 95% CI –0.009 to –0.001; *P*=.008). Authoritative media use increases trust in government, which in turn reduces risk perception, ultimately leading to decreased individual policy compliance behavior, reflecting the phenomenon of the “paradox of trust.” Thus, H3a is supported. By contrast, the chain mediation effect for the path “we-media use→trust in government→risk perception→policy compliance behavior” has a negative path coefficient, but it does not reach statistical significance (β coefficient=–0.001, 95% CI –0.002 to 0.000; *P*=.13). Therefore, H3b is not supported.

## Discussion

### Overview

This study integrates motivation theory and the SARF to examine the impact of media use on public policy compliance behavior during a pandemic. The research aims to reveal the roles of different media in influencing public behavior and the changes in their effectiveness. The results indicate that the public’s use of authoritative media promotes policy compliance behavior by increasing trust in government. By contrast, the use of we-media can indirectly affect individual policy compliance behavior by influencing individual risk perception. The findings also reveal that trust in government and risk perception serve as chain mediators between the use of authoritative media and policy compliance behavior, validating the phenomenon of the paradox of trust.

Specifically, we identified mediating variables that affect individual policy compliance behavior as a result of media use, leading to differences in individual attitudes toward compliance with epidemic prevention policies. This study uncovers the specific mechanisms, or the “black boxes,” through which trust in government and risk perception mediate the relationship between media use and individual policy compliance behavior.

### “Black Box 1”: Normative Motivation (the Bridging Power of Trust in Government)

The study found that trust in government has a substantial mediating effect between the public’s use of authoritative media and policy compliance behavior. However, we-media has no substantial impact on trust in government.

Authoritative media disseminate truthful and objective information, which enhances trust in government [60], thereby increasing the public’s willingness to comply with policies [24,72]. Specifically, authoritative media typically communicate key information such as prevention measures and response strategies with high consistency and clarity. This clear and consistent risk information fills the “information vacuum” caused by the public’s cognitive limitations [7], effectively alleviating information asymmetry. When the public’s need for risk information is satisfied, it fosters a stronger sense of trust in government policies.

In China, policy implementation follows a top-down approach [5], making positive policy perception and trust in the government crucial for individuals to comply with antiepidemic measures and achieve policy effectiveness [26,37]. Looking at examples from different cultural contexts, Swedish citizens exhibit a high level of trust in their government, which makes them more likely to adhere to strategies for managing public health crises, contributing to relatively effective pandemic control [21]. Similarly, studies from Italy indicate that higher levels of trust in government encourage the public to respond more actively to government appeals, resulting in reduced mobility during the pandemic [24]. Therefore, the enhancement of public trust in government can further strengthen the role of authoritative media in promoting individual policy compliance behavior.

The study found that we-media do not substantially influence trust in government, which aligns with previous research [61]. The diverse, emotional, and personalized nature of information on we-media may not be entirely reliable or consistent [16,45]. Furthermore, the decentralized nature of we-media, the rapid cross-regional dissemination of content, and low interpersonal communication costs, combined with limited fact-checking time, increase the likelihood of the spread of false or unverified content [56,57]. This complexity of information may lead to ambiguous or divergent impacts on public trust in government, resulting in we-media not having a substantial effect on trust in government.

### “Black Box 2”: Calculative Motivation (the Impact of Risk Perception)

The study found that the use of we-media can increase the public’s risk perception of diseases, indirectly promoting public policy compliance behavior, consistent with previous research [46]. The results also reveal that the use of authoritative media does not substantially impact individual risk perception.

The public processes risk information obtained through we-media, forming a sense of the significance of the risk, which in turn influences individual risk perception [73]. However, we-media (eg, TikTok and Twitter) may be subject to looser regulation in certain situations, and their accountability mechanisms may not be as robust as those of authoritative media [11]. This increases the likelihood of the rapid spread of misinformation, which may have elevated the public’s risk assessment of the pandemic, thereby further influencing their attitudes toward, and compliance with, antipandemic policies [16]. In addition, the study found that the use of authoritative media does not directly affect individual risk perception, differing from previous research [16]. This may be because, in the Chinese context, authoritative media typically emphasize the accuracy and official nature of information, while during major public health crises, the public may prioritize immediacy and highly perceived risk signals, which are often more easily transmitted through we-media. The information conveyed by authoritative media might be perceived by the public as “official responses” rather than direct threats, thus failing to effectively enhance individual risk perception.

The existence of a mediating effect of risk perception indicates the social amplification of risk by we-media, which can qualitatively and quantitatively amplify signals related to risks, enhance risk perception and risk-related behaviors, and intensify the risks and their consequences [12]. Therefore, the quality and accuracy of information dissemination are crucial for public health, and misleading or inaccurate information can lead to incorrect assessments of risk, subsequently affecting individuals’ policy compliance behavior.

### The Paradox of Trust

The research results indicate that the use of authoritative media does indeed enhance public trust in government, subsequently lowering risk perception and leading to reduced policy compliance behavior, thereby illustrating the paradox of trust. However, we-media has no substantial impact on trust in government.

If the government can implement effective pandemic management strategies and achieve results, people will trust the government’s ability to handle public crises, thereby strengthening its management effectiveness in dealing with pandemics [25,26]. However, there is a complex relationship between public trust in government and individual risk perception. Increased trust in government leading to reduced risk perception may decrease public compliance with preventive policies. If people perceive the risk to be low, they might not feel the need to strictly follow prevention measures such as wearing masks and maintaining social distance [31]. A study on the COVID-19 pandemic in Italy showed that when the public was exposed to authoritative information, their trust in the government increased, but their risk perception decreased, thus reducing their likelihood of complying with preventive policies. This could be because confidence in government institutions can alleviate pandemic-related stress. When people have higher trust in their government, they believe that sufficient measures have been taken to protect them, and they might think it is unnecessary to take personal actions to avoid infection and limit the spread of the virus during a pandemic [27]. The paradox of trust has conflicting effects on policy compliance behavior. The public’s willingness to cooperate is influenced by both their trust in the government and their perceived level of risk. In addition, as mentioned previously, the diversity and complexity of we-media content may lead to confusion and misunderstandings among the public when receiving information, making it difficult to form a consistent view of trust in the government. This uncertainty in the information received may further undermine the role of we-media in shaping trust in government.

### Limitations

The study primarily relied on cross-sectional data, which, while able to describe public psychology and policy compliance behaviors during the data collection period, were limited. The varying stages of the pandemic, changes in government measures, and shifts in public sentiment make trust in policies a dynamic process. Cross-sectional data often fail to capture the lag effects, cumulative impacts, or long-term trends of public health policy outcomes. Future research should consider the long-term effects of dynamic changes in a pandemic on trust and compliance behavior.

Moreover, the limitations of self-reported data, such as social desirability bias and recall bias, may affect respondents’ accurate description of compliance behaviors. Future studies could use more objective measurement methods, such as actual behavior tracking or observational data, to enhance data accuracy. Furthermore, policy compliance behavior is influenced by various factors, including political ideology, media literacy, policy implementation effectiveness, and social opinion, which also warrant further investigation. Subsequent research could introduce these variables to enrich the theoretical framework and test their applicability in different social contexts.

### Conclusions

The study identified the mechanisms through which the use of different media influences policy compliance behavior during a pandemic, as well as the phenomenon of the paradox of trust. Public policy compliance behavior is driven by both normative and calculative motivations. These motivations each tend to rely on different sources of information when guiding public behavior. *Normative motivation* emphasizes individual adherence to social norms, with behavior decisions driven by this motivation being more influenced by authoritative media. Information released by the government, public health agencies, and authoritative media, due to its authority, accuracy, and comprehensiveness, becomes an important benchmark for the public to judge the correctness of their actions. Through the dissemination of authoritative information, public health departments can guide the public to form correct perceptions, enhance trust in the government, and thereby promote policy compliance behavior. However, *calculative motivation* follows a different path. Calculative motivation, based on individual cost-benefit analysis, prompts the public to make behavior decisions after evaluating their own risks and benefits. We-media, such as social media, with their immediacy, interactivity, and personalized features, can quickly spread information that resonates emotionally with the public, potentially promoting policy compliance behavior to a greater extent.

In addition, this study quantitatively analyzes the paradox of trust by constructing a theoretical model. We verified the relationship between trust and compliance behavior through structural equation modeling, confirming that high trust does not necessarily lead to high compliance, thereby clarifying the existence of the paradox of trust. This finding challenges the traditional simplistic assumption of a linear correlation between trust and compliance behavior, providing a new perspective for understanding the relationship between trust and compliance behavior and offering valuable references for future research.

Understanding the paradox of trust can help managers better identify and address trust issues within organizations, as well as optimize organizational structures, incentive mechanisms, and communication methods. By delving into the dynamics of the paradox of trust, public health departments can develop effective and reliable communication strategies to address scenarios where, over time, the public perceives a reduced risk, leading to a decline in willingness to comply with pandemic prevention measures. Such strategies can encourage better adherence to public health policies during pandemics.

The study offers several policy recommendations for public health departments: first, public health officials can enhance public trust by regularly disseminating transparent, evidence-based information. Transparency can reduce uncertainty and prevent the spread of misinformation, thereby helping to improve policy compliance. Second, personalizing and tailoring risk information for different audience groups can help the public better understand the severity of risks and the specific threats they face. Third, the paradox of trust suggests that during a pandemic, governments should focus on enhancing the quality and efficiency of public services to strengthen trust in government. They should also use authoritative media to help the public correctly understand and assess risks, preventing the neglect of risks that could lead to reduced compliance with public policies. Finally, public health departments should establish effective 2-way communication channels to promptly convey risk information, address public concerns, and alleviate panic while avoiding excessive optimism. This balanced guidance of the public’s risk perception can help mitigate the impact of the paradox of trust.

## Abbreviations

CMB: common method bias
EFA: exploratory factor analysis
SARF: social amplification of risk framework
SEM: structural equation model
